# Antibody based conditioning for allogeneic hematopoietic stem cell transplantation

**DOI:** 10.3389/fimmu.2022.1031334

**Published:** 2022-10-20

**Authors:** Asim Saha, Bruce R. Blazar

**Affiliations:** ^1^ Division of Blood & Marrow Transplant & Cellular Therapy, Masonic Cancer Center, University of Minnesota, Minneapolis, MN, United States; ^2^ Department of Pediatrics, University of Minnesota, Minneapolis, MN, United States

**Keywords:** HSCs, hsct, engraftment, immune reconstitution, radioimmunoconjugate, immunotoxin, antibody-drug conjugate

## Abstract

Allogeneic hematopoietic stem cell transplantation (allo-HSCT) is a curative therapeutic option for many patients with hematological malignancies and nonmalignant hematopoietic disorders. To achieve stable engraftment of donor hematopoietic stem cells (HSCs), recipient HSC deletion is needed to create space for incoming donor HSCs and donor HSCs must escape immune rejection by the recipient. Conventional allo-HSCT requires high dose of irradiation and/or chemotherapy to produce sufficient host stem cell and immune system ablation to permit donor HSC engraftment. However, these procedures also result in nonspecific tissue injury that can cause short- and long-term adverse effects as well as incite and amplify graft-versus-host-disease (GVHD). The delivery of targeted radiotherapy to hematopoietic tissues with the use of a radioimmunoconjugate (ROIC) as a part of transplant preparative regimen has shown clinical benefits. ROIC clinical data provide evidence for decreased relapse without increased transplant-related mortality by delivering higher targeted radiation to sites of malignancy than when given in a nontargeted fashion. An alternative approach to allo-HSCT has been developed and tested in preclinical mouse models in which nonmyeloablative preconditioning with low dose of the alkylating agent (busulfan) or lower systemic dose of irradiation combined with co-stimulatory pathway blockade (CTLA4-Ig, anti-CD40L monoclonal antibody) and/or immunosuppressive drugs have been used. Under these conditions, mixed chimerism and transplantation tolerance to fully MHC mismatched donor marrow was observed. Recently, several novel proof-of-concept antibody-mediated preconditioning methods have been developed that can selectively target hematopoietic stem and immune cells with minimal overall toxicity. Antibody-drug-conjugate (ADC) combined with reduced intensity conditioning or high dose ADC as single dose monotherapy have shown promise for allo-HSCT in preclinical models. The purpose of the current review is to discuss the literature exploring antibody-based conditioning that includes native antibody, radiolabeled antibody conjugates, and ADC for allo-HSCT.

## Introduction

Hematopoietic stem cells (HSCs) replenish the blood system throughout the life span of an organism and maintain homeostasis. Hematopoietic stem cell transplantation (HSCT) is an effective treatment modality that enables replacement of host HSCs with HSCs from a healthy donor or genetically corrected HSCs from the patient (1). Although HSCT is most often performed for the treatment of malignancies, HSCT has been successfully employed as treatment of nonmalignant lymphohematological disorders such as thalassemia, sickle cell anemia, aplastic anemia, inherited immunodeficiencies, autoimmune diseases, and metabolic storage disorders (2–10) and for tolerance induction in transplant patients receiving solid organ grafts (11, 12). For engraftment of allogeneic or gene corrected autologous HSCs, two obstacles must be overcome. First, recipient HSCs must be depleted to create niche space for incoming donor HSCs, and second, the transplanted cells must escape immune rejection by the recipient.

HSCT conditioning varies in the degree of myelosuppression and immune suppression from high-dose myeloablative to reduced intensity conditioning (RIC). These conditioning regimens also can result in short-term and long-term complications including multi-organ damage, mucositis, need for frequent red blood cell and platelet transfusions, infertility, and secondary malignancies (13, 14). Allo-HSCT immune complications including multiorgan toxicity associated with conditioning regimens also can incite and amplify graft-versus-host disease (GVHD), limiting the broader array of allo-HSCT applications (15, 16). The choice of RIC depends upon host factors such as age, organ system dysfunction, Hematopoietic Cell Transplantation-specific Comorbidity Index (HCT-CI), Pretransplant Assessment of Mortality (PAM) score, disease risk, GVHD prophylaxis, and donor graft characteristics. For patients with hematological malignancies, the use of RIC is predicated on evidence that the higher-dose regimens may not necessarily offer an advance over RIC in decreasing tumor recurrence, despite the observed increased toxicity (17). While in some nonmyeloablative settings, graft-versus-tumor (GVT) activity was sufficient to diminish or eliminate tumor burden to a comparable extent as myeloablative conditioning, more commonly, lower non-relapse mortality rates were offset by higher relapse rates (18–20). For patients with nonmalignant diseases, the risk of GVHD complications outweighs the potential of a graft-versus-hematopoietic effect that can facilitate alloengraftment (21). As such, there is a considerable interest in finding less toxic myeloablative or RIC conditioning approaches without compromising the positive anti-tumor effects, especially for patients that qualify for RIC and those with nonmalignant disease.

Depending on several donor and host factors and the degree to which RIC regimens eliminate or otherwise incapacitate the host hematopoietic and immune compartments, outcomes can include full or partial chimerism, autologous recovery or aplasia. In situations of partial chimerism, the progressive replacement of host lymphopoiesis and hematopoiesis with the donor’s immune and hematopoietic systems is possible (22). In the absence of donor and endogenous host hematopoietic recovery, graft failure causes a prolonged time period of aplasia and risk of infection due to susceptibility to viral, bacterial and fungal infections. Disease progression may be observed due to lack of donor immune competent cells needed to mount a successful attack on malignant cells that have escaped conditioning.

Myeloablative conditioning regimens include alkylating agents, DNA synthesis inhibitors and/or total body irradiation (TBI) that may serve the dual purpose of treating malignant disorders. For patients with nonmalignant disease, the goals of nonmyeloablative regimens are to prevent rejection of donor cells and open marrow niches of sufficient magnitude and duration to treat the underlying disease while minimizing toxicity. Preclinical studies, including ours, have shown that blocking the CD40/CD40L costimulatory pathway by administering anti-CD40L (anti-CD154) blocking monoclonal antibody (mAb) can effectively induce tolerance in solid organ transplantation models and augment donor bone marrow (BM) alloengraftment in sublethally irradiated recipients of MHC-disparate donor grafts (23–29). In a murine model of sickle cell disease (SCD), recipients conditioned with low dose of the alkylating agent (busulfan) along with anti-CD40L mAb and the fusion protein, CTLA4-Ig that blocks the CD28/CTLA-4 costimulatory pathway became mixed chimeras that permitted the acquisition of normal red blood cell morphology without evidence of GVHD (30). In other studies, mice conditioned with minimal (100-200 cGy) TBI, anti-CD40L mAb, and the mammalian target of rapamycin inhibitor, sirolimus, facilitated alloengraftment and induced profound donor tolerance with uniform donor skin graft acceptance (31).

Engraftment in the complete absence of cytoreductive conditioning also has been possible in mice receiving repetitive infusions of donor BM and anti-CD40L mAb (26) or a very high number of donor BM cells (termed “megadose”) in the context of anti-CD40L mAb and CTLA4-Ig (27); notably, anti-CD40L mAb and CTLA4-Ig are also effective in inhibiting donor anti-host T cell responses culminating in GVHD (25, 32). On the far end of the spectrum, nonirradiated mice given a single high BM dose (80×10^6^ cells), anti-CD40L mAb and sirolimus had macrochimerism levels ranging from 6-15%. With 8 repeated BM doses totaling 160×10^6^ cells (~8×10^9^/kg), anti-CD40L mAb monotherapy in the absence of sirolimus resulted in chimerism levels ranging 6% to 12% (26).

## Naked antibody-mediated cytoreduction to facilitate allo-HSCT

An alternative strategy to safely engraft MHC-mismatched HSCs without chemoradiotherapy would be to employ mAbs that can ablate the recipient’s hematopoietic and immune systems or the hematopoietic system in an immune deficient setting. mAb-based approaches that target CD45 (or c-kit; see below) for recipient HSC depletion have shown promise as non-genotoxic HSCT conditioning agent. As both precursor and mature hematopoietic cells express CD45, anti-CD45 mAb represents an attractive target for HSCT conditioning. CD45 is a membrane glycoprotein whose expression and glycosylation patterns are controlled in a leukocyte-specific manner (33, 34). CD45 is widely expressed in the hematopoietic system, participates in the regulation of lymphocyte activation and maturation, as well as thymic selection (35). All mature leukocytes including tissue-seeded lymphocytes and BM seeded precursor cells express CD45 (35, 36), whereas CD45 dim expression on HSCs has been documented in different species (37, 38). Multiple isoforms for CD45 exist with molecular weights ranging from 180 to 220 kDa (39, 40).

Naked mAbs targeting CD45 have been tested for HSCT conditioning both in mice and human transplantation. In a syngeneic model, a cytolytic anti-CD45 mAb (30F11) treatment alone did not allow donor engraftment. Mice given 5.5Gy TBI and 4×10^7^ BM cells had a mean of ~75% engraftment that did not significantly increase with the addition of the four daily doses of 30F11 mAb. In an allogeneic model in which recipients were given two doses of 2×10^7^ BM, donor chimerism induced by combining anti-CD45 mAb and 8Gy TBI resulted in almost full alloengraftment in marked contrast to mean donor levels of <5% with 8Gy TBI or anti-CD45 mAb alone (38). The lack of adequate conditioning with naked anti-CD45 mAb (30F11) likely occurred as a result of the depletion of lymphoid cells only by host lytic mechanisms, such as complement fixation and/or antibody-dependent cellular cytotoxicity (ADCC), with sparing of myeloid progenitors, necessitating the addition of TBI (41). Similarly, rat anti-human CD45 mAb clones (YTH24.5 and YTH54.12) reduced mature leukocytes and leukemic blasts in BM of patients with acute leukemia but without relevant effects on myeloid precursor cells (41). In an antibody-based minimal-intensity conditioning regimen in patients, anti-CD45 mAbs (YTH24.5 and YTH54.12) along with alemtuzumab (anti-CD52), fludarabine, and low dose cyclophosphamide (Cy) accomplished both myelosuppression and immunosuppression (42). Of 16 high-risk patients with primary immunodeficiency disorders who received allo-HSCT, 15 engrafted, 69% achieved full or high-level mixed chimerism in both lymphoid and myeloid lineages and 19% experienced chimerism but only in the T-lymphoid lineage. For the entire cohort, 81% of this high-risk cohort survived at a median of 40 months (42). Even in these immunodeficiency patients, sufficient alloengraftment with naked anti-CD45 mAb required genotoxic agents.

Signaling engaged by c-kit ligand binding to KIT, a dimeric transmembrane receptor tyrosine kinase expressed by HSCs and their progenitors (43), is essential for HSC homing, proliferation, adhesion, maintenance, and survival (44, 45). The significance of c-kit in HSC regulation can be demonstrated in mildly anemic *W41/W41* mice that have a partial loss of KIT function resulting in reduced HSCs and are readily reconstituted by congenic HSCs with minimal radiation conditioning (46, 47). Administration of ACK2, a blocking anti-mouse c-kit mAb first reported in 2007 targeted reduction in HSCs of sufficient magnitude to allow congenic donor BM engraftment in Rag2^-/-^γc^-/-^ immunodeficient mice (48). ACK2 given *in utero* eliminated fetal HSCs in developing mouse embryos and permitted congenic HSC engraftment in neonates (49). Similarly, significant donor engraftment was seen following HSCT in a mouse model of Fanconi anemia (FA) that has an inherent HSC defect; CD4 depletion and c-kit mAb further improved multilineage donor engraftment in this minor histocompatibility antigen mismatch transplant model as compared to c-kit mAb (50). Whereas ACK2 as a single agent was incapable of conditioning immunocompetent adult mice, adding sublethal TBI permitted meaningful donor chimerism (51). Further, anti-c-kit mAb with low dose of irradiation provided modest long-term engraftment in non-human primates (52).

In other studies, ACK2 and anti-CD47 mAbs were infused into immunocompetent, non-anemic F1 recipients given parenteral donor congenic lineage (neg) BM. At least 50% of HSCs, myeloid cells, B cells and NK cells were of donor origin, while mean donor T cell chimerism was ~30% (53). Here, anti-CD47 Ab worked as a myeloid-specific immune checkpoint inhibitor that blocks a “don’t eat me” signal in monocytes and macrophages (54) to improve ACK2 mediated HSC depletion by ADCC. In a donor and host minor histocompatibility antigen disparate model, mean engraftment levels of ~20% donor HSCs and myeloid cells were seen with ACK2, anti-CD47 mAb, and anti-CD4 plus anti-CD8 mAbs. Without anti-CD47 mAb, ACK2 and anti-CD4 plus anti-CD8 mAbs induced T cell depletion that was insufficient to provide mean donor chimerism levels of >5% (53). To engraft MHC-mismatched HSCs into immunocompetent recipient mice (55), 4 antibodies (anti-CD117, anti-CD47, anti-CD122, and anti-CD40L) were given to haploidentical recipients of nonmanipulated BM cells, resulting in ~20% mean donor chimerism (55). Although transplantation with purified lineage^-^Sca-1^+^c-kit^+^ (LSK) cells failed to engraft, adding anti-CD4 and anti-CD8 depleting Abs improved mean chimerism up to 30%. Engraftment of haploidentical LSK cells induced tolerance to matched heart grafts without loss of transplantation immunity against foreign tissues. All 6 mAbs followed by infusion of donor LSK cells into MHC-mismatched recipients resulted in ~50% mean chimerism, albeit less than mean chimerism levels of >95% in high dose TBI conditioned recipients (55). These data support Ab mediated conditioning for potential broad application in both hematopoietic allotransplantation and tolerance induction in solid organ transplantation.

The myelodysplastic syndromes (MDS), a group of clonal disorders, are characterized by ineffective mature blood cell production and increased risk of progression to acute myeloid leukemia (AML). Successful elimination of MDS HSCs is an important part of curative therapy. The MDS HSC depletion potential of anti-human c-kit mAb (SR-1; CD117) was tested in a xenograft mouse model in which BM from MDS patients was infused. SR-1 and the humanized anti-c-kit mAb, AMG 191, each depleted MDS HSCs in xenografted mice and facilitated engraftment of normal donor human HSCs thereby restoring normal hematopoiesis (56). In non-human primates, injection of a humanized anti-human c-kit mAb, AMG 191 provided measurable hematopoietic stem and progenitor cell depletions (57). Currently a human trial is in progress in children with severe combined immunodeficiency (SCID) using AMG 191 as a conditioning agent to facilitate BM niche clearance before infusion of CD34^+^ enriched grafts (clinicaltrials.gov NCT02963064).

## Radiolabeled antibody as conditioning for stem cell transplantation

Allo-HSCT is the only curative therapy for many patients with advanced AML. However, treatment related toxicity and relapse are still major cause of morbidity and mortality. To increase the radiation dose delivered to the target organs while further reducing the late toxic effects of external beam γ-irradiation, strategies using radioimmunotherapy (RIT) targeted toward hematopoietic tissues as a part of the conditioning regimen have been investigated. RIT, which employs an α-, β- and/or γ-emitting radionuclide conjugated to a targeting Ab, is effective for delivering cytotoxic doses of radiation to a cell type of interest while minimizing off-(tumor) target toxicity. Radiolabeled Abs can target high doses of radiation to lymphoid tissues with at least 2- to 3-fold more radiation delivered to BM, and at least 5-fold more to the spleen and other sites of AML, while sparing normal organs (58–64). CD45 is present at high density on all hematopoietic cells and at least 90% of myeloid leukemias express CD45 (65), making CD45 an attractive target for myeloablative conditioning in patients with AML and MDS.

The myeloablative and immunosuppressive effects of β- and γ-emitter iodine-131 (^131^I) conjugated to an anti-CD45 mAb (30F11) were evaluated in CD45 congenic and H2-mismatched murine marrow transplant models (66). Recipients conditioned with 0.5 mCi ^131^I-anti-CD45 Ab (~17Gy) or 8Gy TBI followed by T cell depleted BM resulted in >80% donor chimerism in a congenic HSCT setting, in contrast to the higher TBI dose (14Gy) that was necessary for allogeneic engraftment. Although MHC-mismatched engraftment occurred in only 3 of 11 mice receiving 1.5 mCi ^131^I-anti-CD45 Ab, engraftment frequency improved significantly in recipients conditioned with 0.75 mCi ^131^I-anti-CD45 Ab along with subablative 8Gy TBI, suggesting radiolabeled Ab can partially replace TBI conditioning (66). The immunosuppressive effects of ^131^I-anti-CD45 Ab were also evaluated in an MHC minor mismatch transplant model (67). T cell depleted BM engraftment could be detected in 86% of the recipients treated with 0.75 mCi of ^131^I-anti-CD45 Ab alone and in 100% of mice treated with either 10Gy TBI alone or 0.75 mCi of ^131^I-anti-CD45 Ab and 2Gy TBI, demonstrating the radiation delivered by 0.75 mCi of ^131^I-anti-CD45 Ab provides a biological effect equivalent to 8Gy TBI (67). Orozco JJ and colleagues have reported that recipients conditioned with a β-emitter yttrium-90 (^90^Y) conjugated to an anti-CD45 mAb combined with pre- and posttransplant Cy in the absence of TBI or fludarabine facilitated high levels of haploidentical BM engraftment and improved survival in a murine leukemia model (68).

α-emitters with short path length and high linear energy transfer could be more suitable for the delivery of highly localized cytotoxic radiation with minimal nonspecific radiation in surrounding tissues as an HSCT conditioning regimen. The α-emitter bismuth 213 (^213^Bi) conjugated to an anti-CD45 mAb (CA12.10C12) was evaluated as a replacement for 2Gy TBI in a canine model of nonmyeloablative dog leukocyte antigen (DLA)-identical marrow transplantation (69). Recipients were conditioned with ^213^Bi-anti-CD45 Ab followed by BM engraftment from DLA-identical littermates and were treated with mycophenolate mofetil combined with cyclosporine for posttransplant immunosuppression. This nonmyeloablative conditioning regimen resulted in stable long-term donor chimerism ranging 30-70% in all recipients. To reduce toxicity associated with external γ-beam radiation, Chen et al. investigated the potential of anti-canine CD45 mAb conjugated with an α-emitter, astatine-211 (^211^At) as a conditioning regimen in DLA identical HSCT (70). Conditioning of recipients with ^211^At-anti-CD45 Ab along with posttransplant immunosuppression consisting of mycophenolate mofetil and cyclosporine resulted stable long-term donor engraftment. The immunosuppressive potential of anti-CD45 mAb conjugated to ^211^At was also evaluated in a canine model of autologous gene transfer. Successful myelosuppression with rapid autologous recovery and minimal off-target toxicity, but only minimal durable engraftment occurred because of a low transduced cell dose (71). In a canine pre-sensitization model using donor blood transfusions, dogs receiving 9.2Gy TBI followed by DLA-identical marrow grafts had graft rejection in 100% of the recipients. However, conditioning of recipients with ^211^At-anti-CD45 Ab along with TBI was successful in abrogating graft rejection in 86% of the recipients (72).

Lytic anti-CD45 mAb or anti-CD45 mAb RIT with standard chemotherapy have been tested in the clinic for conditioning of patients with hematological malignancies with the dual benefit that hematological malignancies are CD45^+^ (63, 64, 73, 74). Inclusion of a ^90^Y-DOTA conjugated to anti-CD45 mAb (BC8), with a standard RIC regimen before allo-HSCT for patients with unfavorable risk multiple myeloma, proved both feasible and well-tolerated (75). This regimen did not result in increased grade ≥ 3 toxicities beyond those expected with alkylating agent and TBI alone (76, 77). Although a maximum dose of 32Gy was delivered to the liver, no grade III/IV dose-limiting-toxicities were observed and no dose related transaminitis was seen, suggesting that patients may tolerate even higher hepatic doses using ^90^Y-anti-CD45 mAb combined with RIC. Toxicity was not increased with ^131^I-anti-CD45 mAb added to myeloablative regimens (58, 60, 64, 78). These examples of targeted RIT deliver high doses to sites of heme malignancies while minimizing other organ toxicities. Notably, anti-CD45 Ab RIT induces neutropenia, lymphopenia, and thrombocytopenia similar to conventional conditioning (63, 70).

## Antibody-drug-conjugate as conditioning for stem cell transplantation

**Figure 1:**
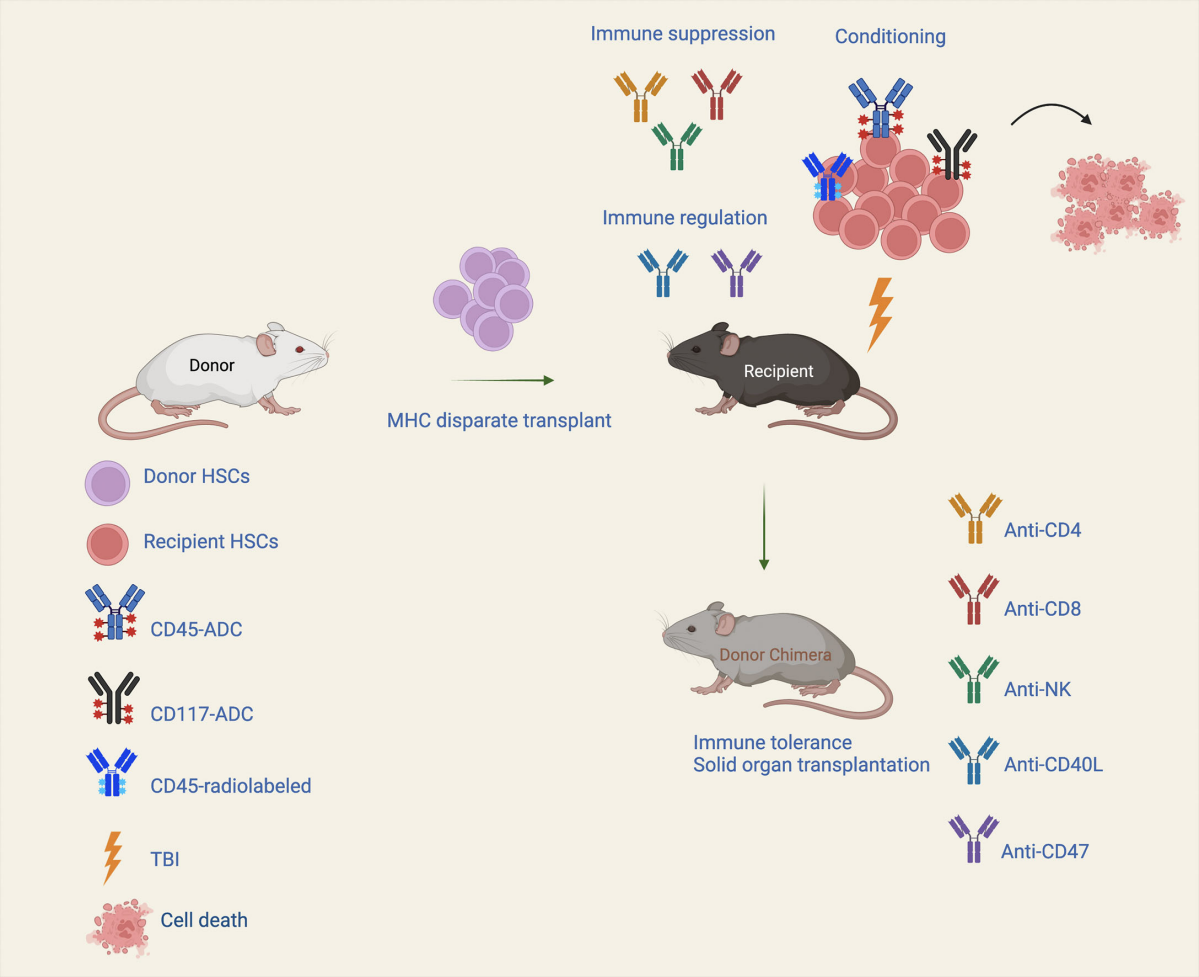
Conditioning approaches for MHC mismatched transplantation. A nonmyeloablative conditioning regimen consist of antibody-drug conjugate (ADC) targeting hematopoietic stem cells (HSCs) along with immune suppression and/or transient immune regulation can enable durable alloengraftment in fully MHC-disparate HSCT recipients. Immunotoxins that can efficiently deplete hematopoietic stem and progenitor cells, and/or lymphoid cells may allow robust donor engraftment with great potential to confer the benefit of fully myeloablative conditioning but with significantly reduced toxicity.

Toxicity associated with chemotherapy or radiation-based conditioning remains a major obstacle for the broader application of HSCT. There is a considerable interest in finding less toxic and more focused approaches to achieve BM conditioning. mAb-based conditioning agents are expected to have much less off-target toxicity than traditional non-targeted modes of cell killing. An alternative mAb-based approach to conditioning involves the use of immunotoxin conjugated to mAbs targeting HSCs, facilitating alloengraftment of transplanted cells while maintaining marrow cellularity (**Figure 1**). Saporin (SAP) and other protein-based immunotoxins have been widely tested in cancer therapy, with greater success for hematological malignancies than solid tumors (79). SAP is a ribosome-inactivating protein with potent cell-cycle-independent cytotoxic activity (80) that can be targeted to specific cell types by conjugation to a mAb directed against cell-surface antigen. SAP is released intracellularly following receptor-mediated internalization resulting in the halting of protein synthesis and induction of cell death (80). Palchaudhuri et al. used anti-CD45 mAb SAP conjugates to deplete HSCs and hematopoietic precursors resulting in durable multilineage donor engraftment (~90%) in immunocompetent mice (81). CD45-SAP conditioning caused a less adverse immediate effect on BM cellularity and preserved thymic function; immune reconstitution appeared early in such recipients as compared to those receiving TBI. Importantly, investigators have shown that pretreatment with a higher dose of CD45-SAP followed by transplantation with congenic BM cells could completely normalize erythropoiesis in a mouse model of human SCD (81). Czechowicz et al. used a similar approach with CD117-SAP as a nonmyeloablative conditioning strategy. CD117-SAP monotherapy enabled robust BM/HSC transplantation in an immunocompetent mouse model avoiding clinically significant collateral damage across tissues (82). The lack of CD117 expression on mature lymphocytes helped maintain immune cell function following treatment with CD117-SAP including retention of effective responses by recipients against both viral and fungal challenges (82). The efficacy of conditioning with CD45-SAP was evaluated in two mouse models of recombinase-activating gene (RAG) deficiency (83). Recipients conditioned with RIC (2Gy TBI) or with CD45-SAP alone resulted in low and intermediate levels of donor engraftment in both the BM and thymus. CD45-SAP with 2Gy TBI resulted in more robust donor engraftment with faster kinetics in both models comparable to conditioning using 8Gy TBI dose. Compared to TBI, CD45-SAP based conditioning allowed significant improvement of thymic architecture and maturation of thymic epithelial cells supporting thymopoiesis (83).

In models of FA and hemophilia A (HA), ADCs were utilized as alternative conditioning regimens (84, 85). FA complement group A (*FANCA*) knockout mice were conditioned with CD45-SAP, CD117-SAP, or Cy followed by transplantation with unmanipulated BM cells from heterozygous healthy donors. ADC conditioning resulted in HSC depletion comparable to Cy treatment, but with substantially less toxicity, and facilitated donor engraftment to levels comparable to Cy conditioning (84). Gao et al. found that a combination of CD45-SAP and CD117-SAP as nongenotoxic preconditioning was effective for factor VIII (2bF8) gene therapy in HA mice. ADC preconditioning permitted long-term engraftment of 2bF8 lentivirus-transduced HSCs, resulting in continued FVIII expression. Supplementing CD8-targeting SAP further improved donor chimerism and FVIII expression in all recipients (85). Although promising, none of these studies evaluated ADC-based nongenotoxic conditioning alone for allo-HSCT (81–85).

Because allo-HSCT is much more challenging to accomplish than syngeneic or congenic HSCT due to the requirement to overcome T cell- and/or NK cell-mediated rejection to enable meaningful engraftment (86, 87), fewer studies have considered ADC-based conditioning for allo-HSCT. Using a fully MHC-mismatched transplantation model, recipients were conditioned with CD117-SAP followed by transient immunosuppression and immune modulation (depleting anti-CD8 mAb, non-depleting anti-CD4 and anti-CD154 mAbs, sirolimus) before transplantation with BM cells (88). Chimerism was not observed following BM transplantation in unconditioned mice, mice conditioned with unconjugated anti-CD117 Ab, or mice conditioned with isotype-control Ab (Iso-SAP). Whereas stable donor chimerism with mean levels of ~50% was observed in 93% of the recipients conditioned with CD117-ADC and transient immunosuppression. Stable chimeras experienced long-term survival of donor-specific skin allograft (88). In another study, using murine allo-HSCT model, Persaud et al. have shown that recipients conditioned with Baricitinib (JAK1/2 inhibitor) and CD45-SAP or CD117-SAP followed by BM transplantation resulted in robust multilineage alloengraftment (89). Unlike TBI-based conditioning, ADC-based conditioning did not promote GVHD alloreactivity in F1 mice challenged with parental splenocytes. Interestingly, using a donor lymphocyte infusion model for graft-versus-leukemia response (GVL), a combination of CD45-ADC and JAK1/2 inhibition provided a balance between tumor control and GVHD outcome as compared to either treatment alone. These data suggest that ADC-based conditioning can provide a suitable platform for the treatment of AML without inducing GVHD (89).

Recently we demonstrated the efficacy of CD45-ADC as a conditioning strategy for multilineage donor engraftment in three distinct mouse transplant models (90). Anti-mouse CD45-ADC, an anti-CD45 mAb engineered for rapid half-life clearance conjugated with a DNA crosslinker payload from the pyrrolobenzodiazepine (PBD) class, effectively promoted alloengraftment. The payload used to develop ADC is tesirine, a PBD dimer that kill cells by antimitotic activity (91). A single dose of CD45-ADC (3 mg/kg) as monotherapy mediated complete depletion of hematopoietic stem and progenitor cells in BM and hematopoietic cells as well as mature lymphocytes in peripheral organs, resulting in robust engraftment in both congenic and minor mismatched transplant models. In an MHC-disparate allo-HSCT model, pretransplant CD45-ADC (3 mg/kg) combined with low-dose TBI and tolerogenic anti-CD40L mAb enabled 89% of recipients to achieve stable alloengraftment. Adding a JAK inhibitor posttransplant further improved alloengraftment. Most strikingly, conditioning fully allogeneic recipients with a single dose of 5 mg/kg or 2 doses of 3 mg/kg CD45-ADC was sufficient for stable multilineage donor engraftment (mean engraftment, >90%) without irradiation or additional immunosuppression, and engraftment potential was comparable to lethal TBI conditioning. Importantly, evidence of potential PBD-related cardiotoxicity was absent by histology. Recipients in all groups remained healthy with almost 100% survival throughout the observation period following allo-HSCT and did not develop any sign of GVHD. Thus, CD45-ADC given pretransplant has the potential utility to confer the benefit of fully myeloablative conditioning but with substantially reduced toxicity when given as a primary agent at a lower dose or at a high dose as monotherapy (90).

We have evaluated the plasma drug concentration of intravenous-administered CD45-ADC versus isotype-ADC over a time course (90). At all doses, CD45-ADC was detectable immediately post infusion, and plasma drug concentration declined rapidly, reaching lower quantification limits by 48 hours. Calculated CD45-ADC clearance and exposure established a substantial target-mediated drug disposition (TMDD) due to a broad CD45 expression profile coupled with a desired rapid half-life (2 to 4 hours for CD45-ADC groups) suitable for juxtaposition to hematopoietic cell infusion. Compared with 3 mg/kg CD45-ADC, isotype-ADC at 3 mg/kg demonstrated a longer half-life (15.3 hours) and higher area under the curve (AUC)-based exposure (3.8-fold), as expected due to lack of TMDD. CD45-ADC mediated depletion of recipient HSCs and mature lymphocytes were routinely evaluated before BMT and based on our findings all HSCT experiments were performed 2-3 days post conditioning (90). In contrast to studies using CD45-SAP mediated conditioning that were performed 7-8 days post conditioning (83–85, 89), the payload used for CD45-ADC was PBD that has a short half-life in mice. Similarly, CD117-SAP mediated depletion of recipient HSCs were evaluated before BMT and HSCT experiments were performed 6-8 days post conditioning (82, 84, 85, 88). Lastly, unconjugated antibodies were also given 6-7 days prior to BMT for HSCT conditioning (48, 50, 53, 55). All these published data suggest that investigators provided sufficient time for clearance of antibodies (conjugated or unconjugated) from serum of recipients before HSCT to avoid antibody mediated elimination of donor stem cells.

For CD45-ADC, there is a broad reactivity and hence depletion of lymphohematopoietic cells would impair host anti-donor immune rejection responses. Whereas CD117 is expressed not only on HSCs but also on a subset of NK cells, common lymphoid progenitors (CLP), common myeloid progenitors (CMP), and prothymocytes that require c-kit for T cell development. CD117-SAP mediated substantial reduction of host HSCs and their progeny through the CLP and CMP differentiation stages overwhelms the host capacity to reject donor HSCs that would have an advantage in competing for BM HSC niches. Further, hybrid resistance accounts for the capacity of parental host NK cells to reject F1 donor BM cells, an immune response that is ineffective when high donor BM cell doses are given to mice. An analogous situation is seen in patients who receive “megadose” transplants and have a higher likelihood of alloengraftment than when conventional BM doses are used.

## Conclusion

Currently, conditioning with alkylating agents alone or with TBI is commonly used in clinical allo-HSCT. These cytotoxic interventions have been deemed useful for patients with malignant diseases. A different situation exists for non-malignant disorders who do not require tumor cytoreduction. Immunotoxin-based approaches (**Table 1**) are receiving greater attention due to lower toxicity compared with chemotherapy or TBI conditions that achieve comparable alloengraftment levels. Recent findings in murine models (53, 55, 81–85, 88–90), nonhuman primates (57, 92), and early human trials (93) support the feasibility and efficacy of antibody and ADC based approaches for cellular therapies. Additional preclinical work will help assess the dosing schedule, safety, age of recipients, the optimal donor cell numbers, the efficacy of other ADCs with superior HSC-depletion potential and degree of off-target specificity. The requirement for receptor-mediated ADC internalization for target cell killing limits the risks of off-target and bystander toxicity. The clinical utility of immunotoxins has been widely tested and safety data available from previous clinical trials can facilitate the clinical translation of immunotoxins for allo-HSCT conditioning. Anti-CD45 mAb RIT continues to have a place as an established strategy for focused elimination of hematopoietic lineage cells. The relative risk:benefit ratio of these approaches (**Table 2**) in the clinic is becoming better understood. For allo-HSCT, ADC-based approaches should significantly reduce major complications and side-effects as compared to standard conditioning regimens and would be more beneficial for patients with nonmalignant disorders including primary immune deficiencies, hemoglobinopathies, inborn errors of metabolism, SCID, and marrow failure syndromes such as Fanconi anemia, where partial donor chimerism can improve the disease condition. Optimization of strategies for allo-HSCT and translation to human clinical trials will be a welcome addition to the armamentarium for treating patients suffering from high-risk malignancies. Anti-human CD117 mAbs and ADCs are under advanced clinical development (93–95). Currently, Magenta Therapeutics is conducting a phase 1 clinical trial of CD117-amanitin for patients with AML and MDS. The successful translation of a mAb-based conditioning approach for MHC-mismatched HSCT would have broad implication for curative treatment across many settings.

**Table 1:** Antibody-based approaches for hematopoietic stem cell transplantation.

HSC targeting agent	TBI	Immune modulation	Drugs	Donor graft	BM / HSC dose	% Chimerism	Organ graft	Reference
*Naked mAb*								
Anti-mouse CD45 (Clone 30F11)	- 5.5Gy - 8Gy	- - Anti-CD4 + Anti-CD8 -	- - - -	Congenic Congenic MHC mismatch MHC mismatch	4×10^7^ BM 4×10^7^ BM 4×10^7^ BM 4×10^7^ BM	<2% (day 30) ~80% (day 30) <5% (day 30) >95% (day 30)	- - - -	(38)
30F11	- 5.5Gy - 8Gy	- - - -	- - - -	Congenic Congenic MHC mismatch MHC mismatch	4×10^7^ BM 4×10^7^ BM 4×10^7^ BM 4×10^7^ BM	<2% (3 months) 65% (3 months) <5% (3 months) >90% (3 months)	- - - -	(41)
Anti-mouse c-kit (Clone ACK2)	–	–	–	Congenic	35×10^3^ HSC	~90% (24 weeks)	–	(48)
ACK2	–	Anti-CD4	–	Minor mismatch	2×10^7^ BM	63% (*Fanca* ^-/-^) (38 weeks) 93% (*Fancd2* ^-/-^) (38 weeks)	–	(50)
ACK2	3Gy	–	–	Haploidentical	1×10^6^ BM	~79% (24 weeks)	–	(51)
*Naked mAb*								
ACK2 ACK2	- -	Anti-CD47 Anti-CD47+ Anti-CD4/8	- -	Haploidentical Minor mismatch	3×10^6^ Lin- BM 15×10^4^ LSK HSC	60% (24 weeks) ~20% (24 weeks)	- -	(53)
ACK2	- - -	Anti-CD47+ Anti-CD122 + Anti-CD40L Anti-CD47+ Anti-CD122 + Anti-CD40L + Anti-CD4/8 Anti-CD47+ Anti-CD122 + Anti-CD40L + Anti-CD4/8	- - -	Haploidentical Haploidentical MHC mismatch	3×10^6^ BM 9×10^3^ LSK 9×10^3^ LSK	~20% (16 weeks) ~30% (16 weeks) >50% (8 weeks)	Accept -	(55)
*RIT*								
^131^I-Anti-CD45 (30F11)	- 8Gy	- -	- -	Congenic MHC mismatch	1×10^7^ BM (T depleted) 1×10^7^ BM (T depleted)	86-94% (16 weeks) (0.5 – 1.5 mCi anti-CD45) >90% (0.75 mCi) (12 wks)	- -	(66)
*RIT*								
^131^I-Anti-CD45 (30F11)	- 2Gy	- -	- -	Minor mismatch Minor mismatch	1×10^7^ BM (T depleted) 1×10^7^ BM (T depleted)	>80% recipients (0.75 mCi) (12 weeks) 100% recipients (0.75 mCi) (12 weeks)	- -	(67)
^90^Y-Anti-CD45 (30F11)	–	–	Cy	Haploidentical	1.5×10^7^ BM	>85% (0.3 mCi) (6 months)	–	(68)
^213^Bi-Anti-CD45 (Clone CA12.10C12)	–	–	MMF + CSP	DLA identical marrow graft	4.7×10^8^ MNCs/kg	30-70% (3.6 – 8.8 mCi/kg) (27 weeks)	–	(69)
^211^At-Anti-CD45 (CA12.10C12)	–	–	MMF + CSP	DLA identical marrow graft	2 – 8×10^8^ MNCs/kg	19-58% (0.2 – 0.6 mCi/kg) (52 weeks)	–	(70)
^211^At-Anti-CD45 (CA12.10C12)	–	–	CSP	Autologous gene modified CD34^+^ cells	0.3 – 2×10^6^ CD34^+^	Low durable engraftment (0.4 – 0.49 mCi/kg)	–	(71)
^211^At-Anti-CD45 (CA12.10C12)	9.2Gy	–	–	DLA identical marrow graft	6.3×10^8^ MNCs/kg	100% (in 86% recipients) (0.2 – 0.4 mCi/kg) (12 months)	–	(72)
*ADC*								
Anti-CD45-SAP (Clone 104)	- -	- -	- -	Congenic Congenic	1×10^7^ BM 2×10^3^ HSC	75-90% (4 months) 90% (4 months)	- -	(81)
Anti-c-kit-SAP (Clone 2B8)	–	–	–	Congenic	1×10^7^ BM	>80% (20 weeks)	–	(82)
104-SAP	2Gy	–	–	Congenic	0.5×10^6^ Lin-	>80% (16 weeks)	–	(83)
2B8-SAP 104-SAP	- -	- -	- -	Heterozygous Heterozygous	1×10^6^ BM 1×10^6^ BM	10-35% (24 weeks) 10-30% (24 weeks)	- -	(84)
2B8-SAP + 104-SAP	–	Anti-CD4/ Anti-CD8	–	Congenic	2.5×10^6^ BM	~75% (20 weeks)	–	(85)
2B8-SAP	–	Anti-CD4/8 + Anti-CD40L	Rapa	MHC mismatch	2×10^7^ BM	~50% (3 months)	Accept	(88)
104-SAP or 2B8-SAP	–	–	JAK1/2 inhibitor	MHC mismatch	1-2×10^7^ BM	~90% (6 months)	–	(89)
*ADC*								
104-ADC (Low dose)	50cGy 50cGy 50cGy	Anti-CD40L Anti-CD40L Anti-CD40L	- Cy/Rapa JAK inhibitor	MHC mismatch MHC mismatch MHC mismatch	4×10^7^ BM 4×10^7^ BM 4×10^7^ BM	54% (12 weeks) 60-77% (12 weeks) 74-78% (12 weeks)	- - -	(90)
104-ADC (High dose)	–	–	–	MHC mismatch	4×10^7^ BM	>90% (22 weeks)	–	(90)

Cy, cyclophosphamide; MMF, mycophenolate mofetil; CSP, cyclosporine; DLA, dog leukocyte antigen; MNCs, mononuclear cells.

SAP, saporin; Rapa, rapamycin; JAK1/2 inhibitor, Baricitinib / ruxolitinib; ADC, antibody-drug conjugate.

**Table 2:** The pros and cons of antibody-based approaches in HSCT.

HSC targeting agent	Advantages	Disadvantages
**Naked mAb**	Non-genotoxic conditioning agent for HSCT	Unconjugated Ab employs relatively weaker host lytic mechanisms such as complement fixation, antibody-dependent cellular cytotoxicity
**Radioimmunotherapy (RIT)**	RIT is effective for delivering cytotoxic doses of radiation to a cell type of interest while minimizing off-target toxicity Radioimmunoconjugates have the potential to decrease relapse without increasing transplant related toxicity by delivering higher doses of radiation to malignant cells	Although radiolabeled anti-CD45 mAbs have been shown to facilitate alloengraftment, logistics and concerns for BM and organ toxicity in allo-HSCT warrant careful consideration. mAb RIT also induce neutropenia, lymphopenia, and thrombocytopenia similar to conventional conditioning
**Antibody-drug conjugate (ADC)**	mAb-based conditioning agents are expected to have much less off-target toxicity than traditional non-targeted modes of cell killing	Transient leukopenia following conditioning with CD45-ADC

## Author contributions

AS wrote the manuscript. BB edited the manuscript. All authors contributed to the article and approved the submitted version.

## Funding

This work was supported in part by National Institutes of Health grants R01 HL147324 and R37 AI34495 and Kids’ First Fund (to BB).

## Conflict of interest

BB receives remuneration as an advisor to Magenta Therapeutics and BlueRock Therapeutics; Rheos Medicines, Childrens’ Cancer Research Fund, and Kids’ First Fund, and is a cofounder of Tmunity Therapeutics.

The remaining author declares that the research was conducted in the absence of any commercial or financial relationships that could be construed as a potential conflict of interest.

## Publisher’s note

All claims expressed in this article are solely those of the authors and do not necessarily represent those of their affiliated organizations, or those of the publisher, the editors and the reviewers. Any product that may be evaluated in this article, or claim that may be made by its manufacturer, is not guaranteed or endorsed by the publisher.
